# BIK1 and ERECTA Play Opposing Roles in Both Leaf and Inflorescence Development in *Arabidopsis*

**DOI:** 10.3389/fpls.2019.01480

**Published:** 2019-11-15

**Authors:** Sufen Chen, Jun Liu, Yu Liu, Lijuan Chen, Ting Sun, Nan Yao, Hong-Bin Wang, Bing Liu

**Affiliations:** State Key Laboratory of Biocontrol, Guangdong Provincial Key Laboratory of Plant Resources, School of Life Sciences, Sun Yat-sen University, Guangzhou, China

**Keywords:** BIK1, ERECTA, inflorescence architecture, leaf morphogenesis, plant development

## Abstract

Plants employ cell-surface receptor-like kinases to detect extrinsic and intrinsic signals, thus make a trade-off between growth and immunity. The receptor-like cytoplasmic kinases on the cytoplasmic side act as downstream components involved in the activation, transmission, and integration of intracellular signals. In *Arabidopsis thaliana*, the RLCK BOTRYTIS-INDUCED KINASE1 (BIK1) associates with multiple RLKs to regulate pathogen defense responses and brassinosteroid (BR) signaling. However, little is known about the biological functions of BIK1 in developmental processes in Arabidopsis. In this study, we established that mutation of ERECTA (ER), an important RLK, counteracts the developmental effects of loss of BIK1 function. BIK1 and ER play opposing roles in leaf morphogenesis and inflorescence architecture. Moreover, we confirmed that BIK1 is required to maintain appropriate auxin response during leaf margin morphogenesis. Finally, we found that BIK1 interacts with ER-family proteins and directly phosphorylates ER. Our findings might provide novel insight into the function of BIK1 in leaf and inflorescence development.

## Introduction

Plants use cell-surface-localized receptor-like kinases to perceive diverse signals, such as plant-derived sterols and peptides, and pathogen-derived molecules (Shiu and Bleecker, 2001), which trigger complex cellular networks with distinct signaling outputs (Belkhadir et al., 2014). For example, the leucine-rich repeat RLK BRASSINOSTEROID-INSENSITIVE1 perceives brassinosteroids (BRs) (Kinoshita et al., 2005). The RLKs CLAVATA1 and ERECTA recognize the CLAVATA3 peptide and epidermal patterning factors (EPFs)/EPF-like proteins (EPFLs), respectively (Ogawa et al., 2008), all of which play essential roles in plant growth and development. In addition, FLAGELLIN-SENSITIVE2 (FLS2) and EF-TU receptor detect bacterial flagellin (or flg22 peptide) and elongation factor Tu (or elf18 peptide) respectively, which modulate plant immune signaling (Gomez-Gomez and Boller, 2000; Zipfel et al., 2006). The coordination of growth and immunity signaling with specific receptor–ligand interactions effectively improves the ability of plants to adapt to environmental changes.

*ERECTA*, which encodes a LRR receptor-like Ser/Thr kinase (Torii et al., 1996), functions synergistically with its homologs, *ER-LIKE1* and *ERL2*, to regulate diverse aspects of plant development and responses to environmental changes, including stomatal formation and patterning (Shpak et al., 2005; Lee et al., 2015; Meng et al., 2015), transpiration efficiency (Masle et al., 2005), inflorescence architecture (Shpak et al., 2004; Woodward et al., 2005; Cai et al., 2017), ovule development (Pillitteri et al., 2007), leaf morphogenesis (Tameshige et al., 2016), and responses to biotic and abiotic stresses (Llorente et al., 2005; van Zanten et al., 2009; Shen et al., 2015; Jorda et al., 2016). The ER loss-of-function mutant *er-105* displays abnormal developmental phenotypes, such as toothless leaves, reduced plant height, and compact inflorescences with shortened internodes, siliques, and pedicels (Clark et al., 1997; Shpak et al., 2003; Tameshige et al., 2016). In ER signaling, ER receptor family members recognize several secreted EPF or EPFL peptides to modulate multiple biological processes. For example, members of the ER family recognize EPF1, EPF2, and EPFL9 to control stomatal patterning (Shpak et al., 2005; Hunt et al., 2010; Lee et al., 2012), whereas EPFL4 and EPFL6 have been identified as ligands of ER in the regulation of inflorescence architecture (Abrash et al., 2011; Uchida et al., 2012), and EPFL2 is recognized by ER-family proteins to control the development of leaf serrations (Tameshige et al., 2016). Signals perceived by ER-family proteins are then delivered to a downstream mitogen-activated protein kinase cascade composed of YODA, MPKK4/5, and MPK6/3 to regulate gene expression (Meng et al., 2012). Interestingly, ER may crosstalk with other signaling pathways at different levels. BRASSINOSTEROID-INSENSITIVE2, a negative regulator of BR signaling, also regulates stomatal development *via* phosphorylating several components downstream of the ER signal pathway, such as YDA, MKK4/MKK5, and SPEECHLESS (Gudesblat et al., 2012; Kim et al., 2012; Khan et al., 2012). However, the molecular mechanism that underlies how ER transmits signals to control the development of leaf and inflorescence remains unclear.

Instead of ligand perception, receptor-like cytoplasmic kinases in the cytoplasm often complex with RLKs and act as downstream components in the activation, transmission, and integration of intracellular signals (Macho and Zipfel, 2014). In *Arabidopsis thaliana*, BOTRYTIS-INDUCED KINASE1 belongs to the RLCK family and play essential roles in immune signaling and BR signaling (Lu et al., 2010; Lin et al., 2013; Li et al., 2014; Lal et al., 2018). In immune signaling, FLS2 or EFR forms a ligand-induced complex with BRI1- ASSOCIATED RECEPTOR KINASE1, a well-studied RLK that often acts as a coreceptor. The BIK1 protein associates with the FLS2/EFR and BAK1 complex and is directly phosphorylated by BAK1 (Lu et al., 2010; Zhang et al., 2010). Following ligand perception, BIK1 dissociates from the complex and activates the downstream nicotinamide adenine dinucleotide phosphate oxidase RESPIRATORY BURST OXIDASE HOMOLOG isoform D and riggers a burst of reactive oxygen species that plays a positive role in plant immunity (Lu et al., 2010; Zhang et al., 2010; Lin et al., 2013; Li et al., 2014). In addition, BIK1 protein is a negative regulator of BR signaling: in the absence of BRs, BIK1 associates with BRI1 and inhibits the initiation of BR signaling, and following BR treatment, BIK1 is released from association with BRI1 and is phosphorylated to transduce BR signaling (Lin et al., 2013).

Although it is well known that BIK1 mediates opposite functions in plant immunity and BR signaling, studies have not fully characterized the biological function of BIK1 in regulating growth and development. For example, the BIK1 loss-of-function mutant *bik1* displays abnormal developmental phenotypes, such as leaves that occasionally curl, with serrated margins and wrinkled surfaces, weaker stem strength, reduced standing ability and lodging, loose inflorescences, reduced fertility, and smaller siliques (Veronese et al., 2006). Moreover, the BIK1 complementation plants rescue the growth defects in the *bik1* mutants, including early flowering, and twisted and curling rosette leaves (Lin et al., 2014; Lal et al., 2018). These developmental features suggest that BIK1 is developmentally important for leaves and inflorescences. However, the mechanism of BIK1 functions in these developmental processes remains unclear, as it is neither mediated by BR signal nor immune related (Lin et al., 2013; Liu et al., 2017).

In this study, we performed an ethyl methanesulfonate mutant screen for modifier of *bik1* developmental phenotypes (*mobd*) to investigate the mechanism of BIK1 in development regulation. Mutants that rescued the serrated leaf-margin and loose inflorescences of *bik1* were identified. We demonstrate that ER is essential for BIK1-mediated developmental phenotypes. BIK1 interacts with ER-family proteins and might function under the presence of ER in leaf morphogenesis and inflorescence architecture. Moreover, our data confirm that ER and BIK1 are both required for auxin responses in the leaf margin and appear to play opposing roles during leaf tooth development.

## Materials and Methods

### Plant Materials and Growth Conditions

The *A. thaliana* Columbia-0 ecotype was used as the wild-type. The following mutants have been described previously: *bik1* (Veronese et al., 2006), *er-105* (Torii et al., 1996). The double mutants *er-120 bik1* (*mobd1 bik1*) and *er-121 bik1* (*mobd2 bik1*) were created through EMS mutagenesis, and by crossing them with the WT plant respectively, we obtained the *er-120* (*mobd1*) and *er-121* (*mobd2*) single mutant. The *er-105 bik1* double mutant was generated by genetic crossing and confirmed by genotyping. For cultivation on soil, plants were grown in a growth chamber at 22°C, 60% relative humidity, and 110 µmol m^−2^ s^−1^ light under photoperiods of 12 h light and 12 h dark. To grow seedlings in sterile culture, seeds were surface-sterilized for 1 min in 70% ethanol and 20% bleach for 10 min, washed 10 times thoroughly in sterile water and then sown on half-strength Murashige and Skoog (1/2MS) medium.

### Ethyl Methanesulfonate Mutagenesis and Isolation of *er* Mutants

The *er-120 bik1* (*mobd1 bik1*) and *er-121 bik1* (*mobd2 bik1*) were isolated from an EMS-mutagenized *bik1* mutant seeds as described (Boerjan et al., 1995). The M_2_ seeds were harvested and screened for the lines which showed modification of *bik1* developmental phenotypes (*mobd bik1*), and the transmission of the phenotype was confirmed in the M_3_ generation. Subsequently, the *mobd1 bik1* and *mobd2 bik1* were crossed with WT plant respectively, single mutant *mobd1* or *mobd2* of EMS mutagenesis were isolated and mapped with SNP-mediated sequencing performed by Shanghai Biotechnology Corporation (Shanghai, China).

### Reverse Transcription-Coupled Polymerase Chain Reaction

Total RNA was extracted from Arabidopsis rosette leaves using the RNeasy Plant Mini Kit (Qiagen). The RNA samples were reverse transcribed into first-strand cDNA using the PrimeScript RT Reagent Kit (TaKaRa, Dalian, China). Both random and oligo(dT)-containing mix primers were used to reverse transcribe the first-strand cDNA. *UBIQUITIN10* was used as the reference gene. The primer sequences are listed in **Supplementary Table S1**.

### Superimposition of Leaf Outlines and Quantification of Leaf Tooth Growth Level

The leaf images were acquired by a stereomicroscope after bleaching in ethanol. Then the petiole part was removed to generate blade-only images as shown in **Figure 4B**. The superimposition of leaf outlines was performed using R language as previously described (Tameshige et al., 2016).

The “solidity” and “tooth height/width ratio” were measured from the binary blade-only images used for the outline superimposition by ImageJ software. The values of Tooth growth level were quantified as “1-solidity” as previously described (Tameshige et al., 2016). To measure Tooth Height/Width ration, the second teeth on the left and right sides were used, and the calculation method was as previously described (Tameshige et al., 2016).

### Microscopy Characterization of DR5pro::GFP

The *DR5pro::GFP* line expressing the auxin-responsive green fluorescence protein (GFP) reporter was described previously (Benkova et al., 2003). The *bik1* and *er-105* mutants were crossed to *DR5pro::GFP*, and homozygous transgenic plants were selected. The newly initiating leaves, the fifth leaves of 16-day-old plants or the seventh leaves of 21-day-old plants were analyzed for GFP fluorescence. Confocal images were captured using a Zeiss LSM780 confocal laser scanning microscope with the following excitation and emission wavelengths : 488 nm/505 to 530 nm for GFP. All images were captured with the same detector settings for GFP fluorescence. The images were converted into binary images under the identical exposure on ImageJ software. And the GFP intensity in the tooth periphery regions (framed by a dashed rectangle of the same size) was quantified using the Measure menu.

### Histochemical Analysis and Microscopy

To characterize of the *BIK1* and *ER* gene expression pattern, 1,800-bp sequence upstream of the ATG codon of the *BIK1* or *ER* gene was amplified with specific primers (**Supplementary Table S1**). The promoter was cloned into pCAMBIA1381Z to drive the expression of the *GUS* (*β*-glucuronidase) reporter gene. Histochemical GUS staining was performed as described previously (Jefferson et al., 1987). Images were captured under a stereomicroscope.

Scanning electron microscopy on silique tips was performed as described by Shpak et al, (2003). The length of silique tips was measured by ImageJ software.

Fixation, embedding, and sectioning of tissues for light microscopy using a stereomicroscope were performed as described by Uchida et al. (2012). The average cortex cell length and total cortex cell number of each pedicel were measured by ImageJ software.

### Coimmunoprecipitation Assay

For coimmunoprecipitation (co-IP) assays, 4 ml Arabidopsis protoplasts was cotransfected with 400 µg *ER/ERL1/ERL2-FLAG* and 400 µg *BIK1-HA* plasmids or transfected with 400 µg *BIK1-HA* plasmid alone. Total proteins were extracted with 500 µl protein extraction buffer and 20 µl protein extract was used as the input fraction. The remaining extracts were further incubated with 30 µl anti-FLAG M_2_ affinity gel for 3 h at 4°C with gentle rotation. After washing three times with ice-cold TBS buffer (pH 7.4), the bound proteins were eluted by boiling the gel using 30 µl SDS-PAGE (dodecyl sulfate sodium salt-Polyacrylamide gel electrophoresis) sample buffer without *β*-mercaptoethanol and were loaded onto SDS-PAGE gels for immunoblotting.

### Recombinant Protein Expression and Purification

The full-length of ORF of BIK1 was cloned into the pET-28 vector or pGEX-4T-1 vector to express the His-tagged and GST-tagged recombinant protein, respectively. Truncated ER, ERL1, or ERL2 with cytoplasmic domain (CD) were introduced into the pGEX-4T-1 vector to express the GST-tagged recombinant protein, and truncated ER with CD was introduced into the pMAL-5cx vector to express the MBP-tagged recombinant protein. His-, GST-, or MBP-tagged recombinant proteins were expressed in the *Escherichia coli* BL21 strain and purified with Ni-Sepharose beads (GE Healthcare, UK), Glutathione Sepharose beads (GE Healthcare, UK) or Amylose Resin (New England Biolabs, USA), respectively, according to the manufacturers’ instructions. The primer sequences are listed in **Supplementary Table S1**.

### *In Vitro* Pull-Down Assay

For *in vitro* pull-down assay, 10 µg BIK1-His and 10 µg GST-ER/ERL1/ERL2-CD or GST were incubated with 30 µl glutathione agarose beads in a buffer containing 25 mM Tris-HCl (pH 7.5), 150 mM NaCl, and 1 mM DTT for 3 h. The beads were washed seven times with the washing buffer containing 25 mM Tris-HCl (pH 7.5), 150 mM NaCl, 1 mM DTT, and 0.1% Trition-X 100. The bound proteins were eluted with an elution buffer containing 25 mM Tris-HCl (pH 7.5), 150 mM NaCl, 1 mM DTT, and 30 mM glutathione, and BIK1-His was detected by immunoblotting with anti-His antibody (Genscript, China).

### *In Vitro* Kinase Activity Assay

The *in vitro* kinase activity assay was performed as previous described (Zhou et al., 2019). In brief, reactions were performed in 30 µl of kinase buffer [20 mM Tris-HCl at pH 7.5, 10 mM MgCl_2_, 5mM EGTA, 100 mM NaCl, and 1 mM dithiothreitol containing 10 µg fusion proteins with 0.1 mM cold ATP and 5 µCi (^32^P) γ-ATP] at room temperature for 3 h with gentle shaking. The reactions were stopped by adding 4× SDS loading buffer. The phosphorylation of fusion proteins was analyzed by autoradiography after separation with 12% SDS-PAGE gels.

## Results

### ER Mutation Modified the Developmental Phenotypes of *bik1* Mutant

**Figure 1:**
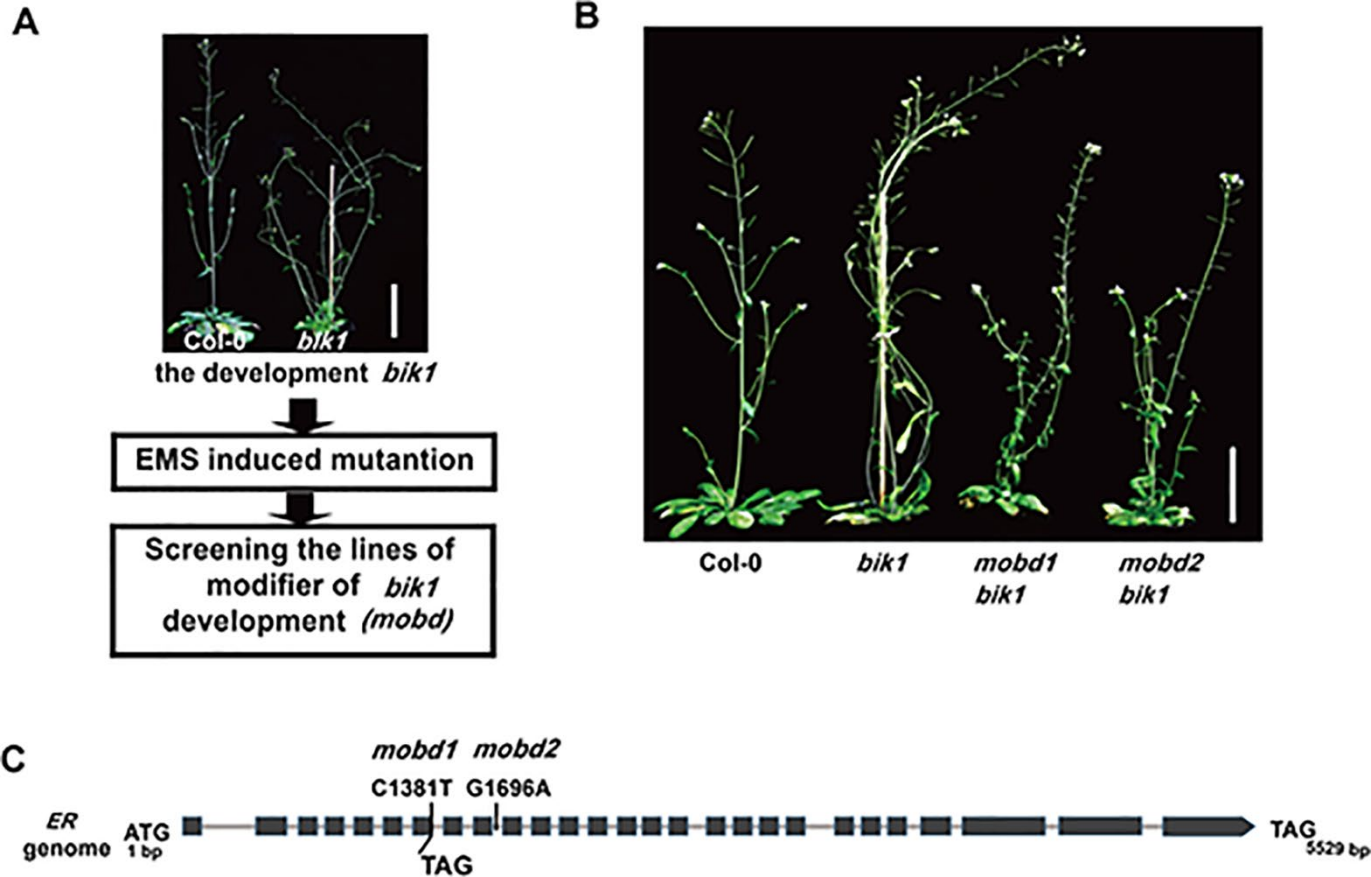
The ERECTA (*ER*) mutation modifies BOTRYTIS-INDUCED KINASE1 (BIK1)–mediated developmental phenotypes. **(A)** The process to screen the modifier of *bik1* development (*mobd*) by ethyl methanesulfonate (EMS) induced mutation. **(B)** The *mobd1 bik1* and *mobd2 bik1* mutants showed suppressed lodging and loose inflorescences triggered by EMS induced mutation. Mature phenotypes of the 9- to10-week-old plants. Scale bar = 5 cm. **(C)** The *ER* gene were edited in *mobd1* and *mobd2*. Total DNA was extracted from the *mobd1* and *mobd2* mutants, the mutated sites of *ER* gene were detected by polymerase chain reaction amplification and sequencing.

Receptor-like cytoplasmic kinase BIK1 is required for normal plant growth and development, in addition to its roles in immune responses and BR signaling (Veronese et al., 2006; Lin et al., 2013; Liu et al., 2017). In order to elucidate the molecular mechanism that underlies the developmental phenotypes of *bik1*, we carried out an EMS-based genetic screen of mutants which modified the phenotypes of *bik1* and named as *modifier of bik1 developmental phenotypes* (*mobd*) (**Figure 1A**). In the M2 generation, we identified two individual mutant lines showed the same phenotypes that largely rescued the lodging and loose inflorescences of *bik1*, named as *mobd1 bik1* and *mobd2 bik1* (**Figure 1B**).

To assess which gene mutation is the cause of the suppressed developmental defects in *mobd1 bik1* and *mobd2 bik1*, we crossed the *mobd1 bik1* and *mobd2 bik1* mutants with Columbia-0 wild-type plant, respectively, and screened the single mutant of *mobd1* and *mobd2* in the F2 generation that were stable inheritance in F3 generation. By SNP (single nucleotide polymorphism)-based whole-genome deep sequencing, we found that both *mobd1* and *mobd2* carried a mutation in *ER* gene, as a C1381-to-T mutation in *mobd1* resulting in the conversion of 191th amino acid in the sixth LRR domain from Gln to a premature stop codon, and a G1696-to-A mutation in the ninth intron of *ER* gene in *mobd2* (**Figure 1C**), similar result was obtained with polymerase chain reaction (PCR) amplification and sequencing. Furthermore, the suppression of *ER* expression in *mobd2* was verified by RT-PCR (reverse transcription–PCR) (**Supplementary Figure S1**). These results suggest that both *mobd1* and *mobd2* were *ER* mutants, and was subsequently named as *er-120* and *er-121*, respectively.

### *bik1* and *er* Mutants Display Opposite Developmental Phenotypes in Terms of Plant Architecture and Inflorescence Development

**Figure 2:**
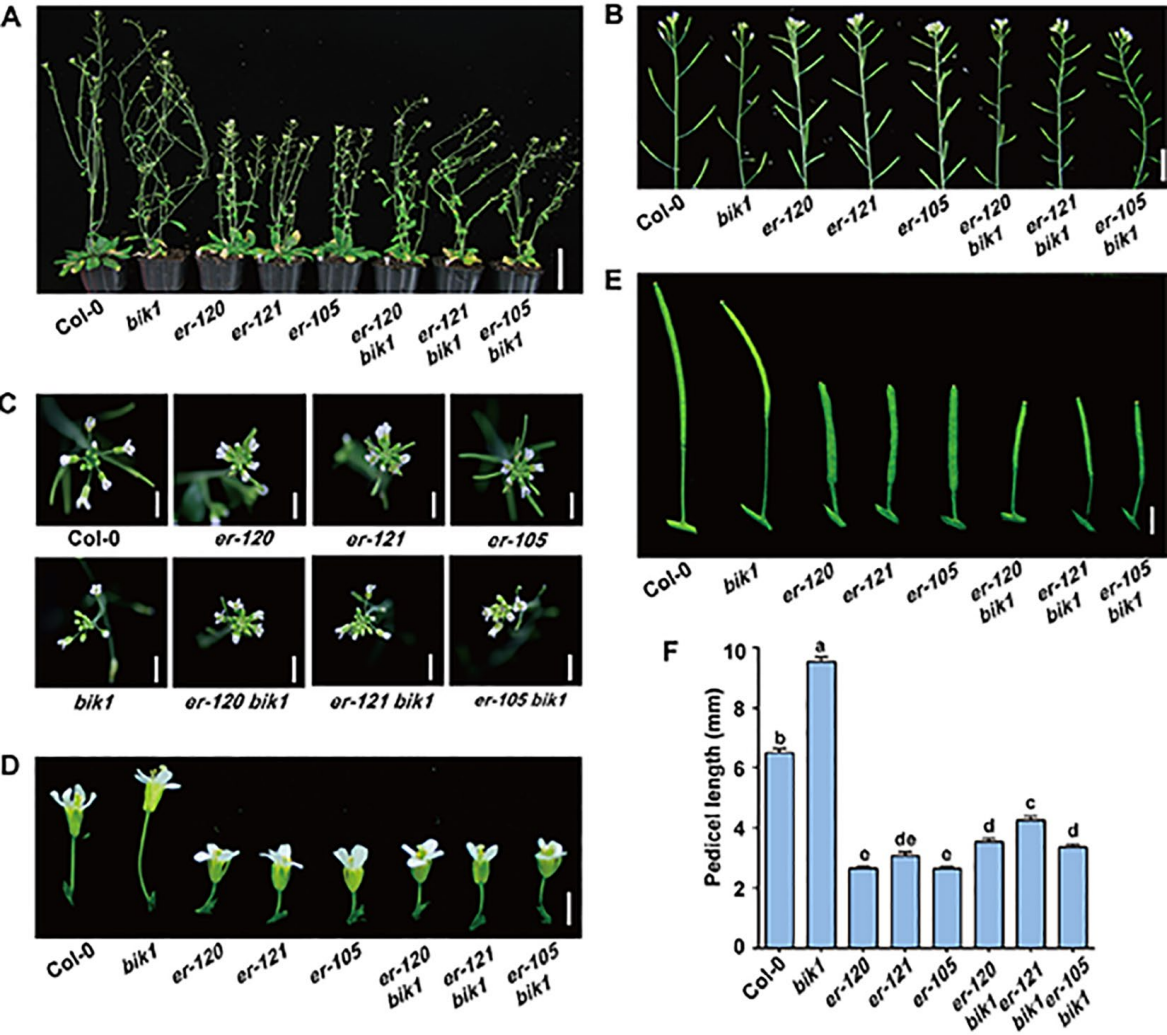
*bik1* and *er* show opposite phenotypes in terms of plant architecture and inflorescence development. **(A)** The growth phenotypes of the 9- to 10-week-old mature plants. Scale bar = 5 cm. **(B)** Inflorescence architecture of 8-week-old plants. Scale bar = 1 cm. **(C)** Architecture of inflorescence stem apices of 8-week-old plants. Scale bar = 0.5 cm. **(D)** Fully open mature flowers and attached pedicels from 8-week-old plants of the respective genotypes. Scale bar = 0.5 cm. **(E)** Siliques and attached pedicels of the 8-week-old plants. Scale bar = 0.5 cm. **(F)** Lengths of mature pedicels on the main stems of fully open flowers from 8-week-old plants. Bars represent mean values ± SEM (n = 10, one pedicel on the main stem per plant with 10 plants per genotype). Different letters indicate significant difference at *p* < 0.05, as determined by one-way ANOVA with Tukey’s post-test.

The *er-120*, *er-121*, and previously reported *er-105* mutant plants all displayed a characteristic compact inflorescence with shortened internodes and pedicels (**Figures 2A–F**), consistent with previous reports that ER promotes inflorescence elongation (Shpak et al., 2003). Conversely, the BIK1 loss-of-function mutant *bik1* exhibited lodging phenotypes, with loose inflorescences, elongated internodes, and pedicels, compared to Col-0 (**Figures 2A–F** and **Supplementary Figure S2**) (Veronese et al., 2006). These findings suggest that BIK1 plays an opposite role to ER in plant architecture and inflorescence development.

To further investigate the functional relationship between ER and BIK1, we generated the *er-105 bik1* double mutant by genetic cross and observed its developmental phenotypes. Interestingly, *er-105 bik1* double mutant showed similar average internodes length compared to that of Col-0 (**Supplementary Figure S2**), suggesting a rescue of internodes length in *er-105 bik1* double mutant. Moreover, although *er-120 bik1*, *er-121 bik1*, and *er-105 bik1* double mutant possessed a compact inflorescence and shorted pedicels compared to Col-0 (**Figures 2C–F**), a statistically significant elongation of pedicels length was observed in these double mutant lines, compared to *er* single mutant lines (**Figures 2C–F**), suggesting a partial rescue of inflorescence development in *er bik1* double mutant lines.

### *bik1* Mutant Displays Altered Silique Tips and Pedicel Cell Proliferation

**Figure 3:**
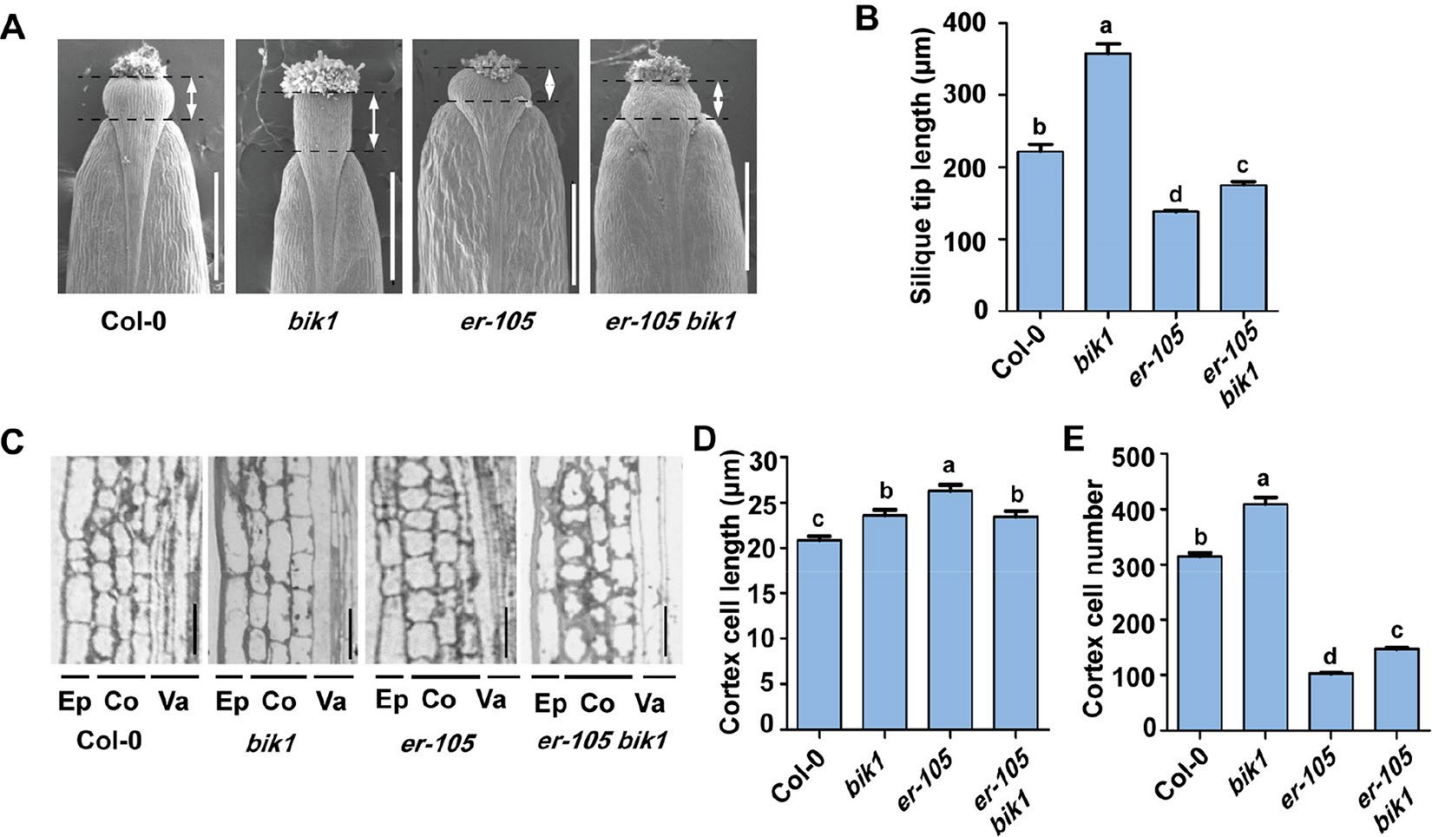
The *bik1* mutant displays altered silique tips and pedicel cell proliferation. **(A)** Scanning electron micrographs of silique tips from 8-week-old plants. Scale bar = 500 μm. **(B)** Quantitative analysis of silique tip length in **(A)**. Double arrows indicated the silique tip regions for measurement. Bars represent mean values ± SEM (n = 10, one silique per plant with 10 plants per genotype). **(C)** Longitudinal sections of mature pedicels from fully open flowers of 8-week-old plants. Ep, epidermis; Co, cortex; Va, vasculature. Scale bar = 25 μm. **(D)** Quantitative analysis of cortex cell length in **(C)**. Bars represent mean values ± SEM (n = 20, two sections per plant with 10 plants per genotype). **(E)** Cell number in the longitudinal cortex file of a mature pedicel from a fully open flower. Bars represent mean values ± SEM (n = 10, one pedicel on the main stem per plant with 10 plants per genotype). Different letters indicate significant difference at *p* < 0.05, as determined by one-way ANOVA with Tukey’s post-test.

The loss-of-function mutant *er-105* plants had shortened, blunt siliques, and the silique tips were also short and broad (Shpak et al., 2003). We also analyzed the function of BIK1 in the morphology of the silique tips in detail (**Figures 3A, B**). The silique tips of Col-0 plants have an elongated and narrow style, whereas the silique tips of *bik1* plants were longer and the valves were narrower than that of Col-0 (**Figures 3A, B**). Furthermore, the silique tips of *er-105 bik1* double mutants were longer than that of *er-105* plants, suggesting a partial suppression of the silique phenotypes of *er-105* single mutant (**Figure 3B**).

To analyze the *bik1* inflorescence defects at the cellular level, we further sectioned pedicel tissue from Col-0, *bik1*, *er-105*, and *er-105 bik1*, and found that *er-105* pedicels contained large and expanded cortex cells but fewer cortex cells number as previously reported (**Figures 3C–E**) (Shpak et al., 2003). In contrast, *bik1* pedicels contained more cortex cells number and elongated cells compared to Col-0, which caused an increase in pedicel length (**Figures 2F** and **3C–E**). Moreover, the *er-105 bik1* double mutant plants showed a reduction in cortex cell length, but an increment in cortex cell number compared to *er-105* mutant (**Figures 3D, E**). This finding revealed that the elongation of pedicels in *er-105 bik1* compared to *er-105* is mainly due to the increment of cortex cell number (**Figures 2F** and **3D, E**), suggesting BIK1 and ER have opposite functions in the pedicel cells proliferation.

### *bik1* and *er* Show Opposite Leaf-Margin Phenotypes

**Figure 4:**
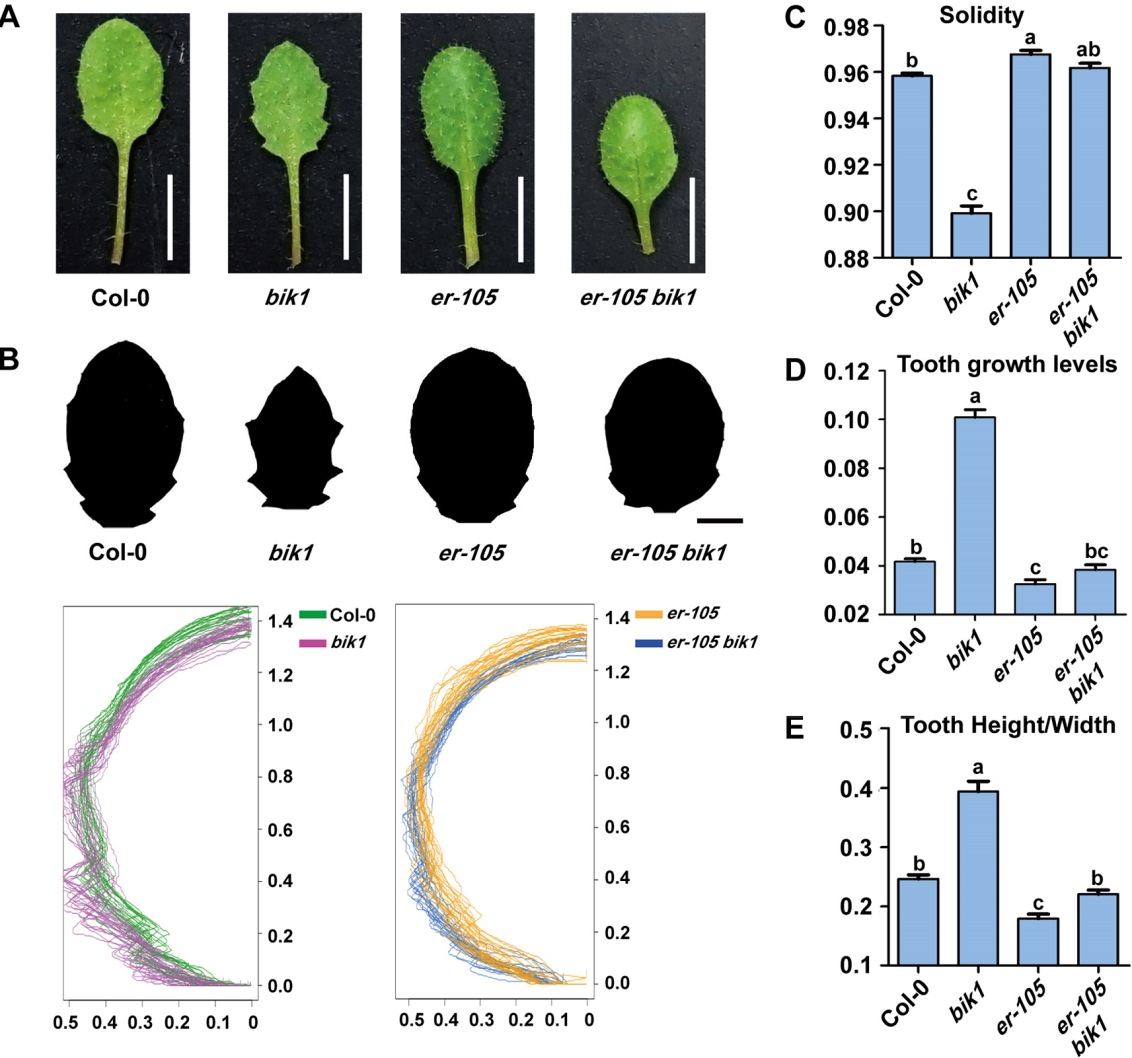
*bik1* and *er* show opposite leaf margin phenotypes. **(A)** The mature eighth leaf of 3-week-old plants. Scale bar = 5 mm. **(B)** Black-and-white images of the eighth leaf from 3-week-old plants. Scale bar = 2 mm. Superimposition of leaf outlines of the eighth leaves from 3-week-old plants. n = 12. The *X* and *Y* values of each leaf are scaled proportionally so that each leaf size is 1; thus, the size of half leaves shown is 0.5. **(C**–**E)** Quantitative measurement of solidity **(C)**, tooth growth levels **(D)**, and tooth height/width **(E)** from the binary blade-only images used for the outline superimposition. Bars represent mean values ± SEM (n = 12). Different letters indicate significant difference at *p* < 0.05, as determined by one-way ANOVA with Tukey’s post-test.

The *bik1* mutant displayed wrinkled and curled leaves with serrated margins, which grows at the later developmental stage, whereas mutation of *ER* caused an opposite phenotype of toothless leaves with smooth margins (**Figure 4A**). To further analyze the shape of *bik1* mutant leaves, we superimposed and compared the outlines of Col-0, *bik1*, *er-105*, and *er-105 bik1* leaves. Tooth outgrowth in *bik1* mutant leaves was greater than that in Col-0 leaves, whereas reduced tooth outgrowth was observed in the leaves of *er-105* compared to Col-0 (**Figure 4B**). To quantify tooth growth phenotypes, we applied several quantification methods: solidity, tooth growth levels (1-solidity), and tooth height/width ratio (Tameshige et al., 2016). The *bik1* and *er-105* possessed opposite leaf-margin phenotypes as shown in **Figures 4C–E**. Moreover, the *er-105 bik1* double mutant plants showed the leaf-margin phenotypes similar to that of Col-0, including the leaf outlines and tooth height/width ratio (**Figures 4B, E**). This indicates that BIK1 and ER both contribute to plant leaf-margin morphogenesis but play opposite role.

### BIK1 is Involved in Auxin Responses During Leaf-Tooth Growth

**Figure 5:**
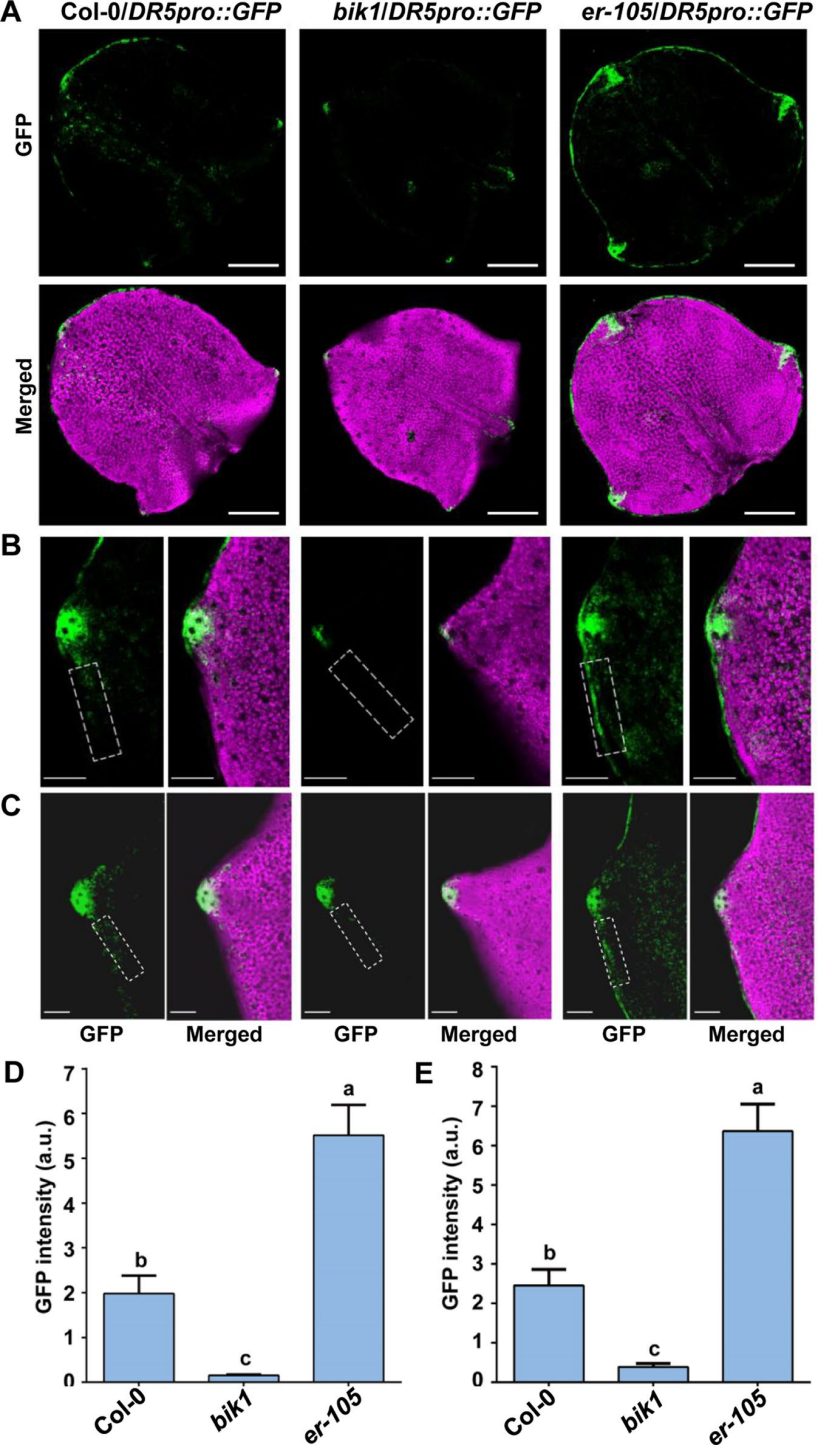
BOTRYTIS-INDUCED KINASE1 (BIK1) is involved in auxin response during leaf-tooth growth. **(A)** Z-projected confocal micrographs of newly initiating leaves in Col-0, *bik1*, and *er-105* plants. Response to auxin is indicated by *DR5pro::GFP* (green) expression. The leaf shape is shown by chlorophyll fluorescence (magenta). Scale bar = 200 μm. **(B**–**C)** Z-projected confocal micrographs of growing tooth in Col-0, *bik1*, and *er-105* leaves. The fully spreading out fifth leaves of 16-day-old plants **(B)** and the fully spreading out seventh leaves of 21-day-old plants **(C)** were analyzed. Dashed rectangles indicate the tooth periphery regions used to measure the GFP intensity in **(D)** and **(E)**. Scale bar = 100 μm. **(D**, **E)** Bar plots of GFP fluorescence intensity in **(B)** and **(C)**, respectively. Bars represent mean values ± SEM (n ≥ 12 per genotype). Different letters indicate significant difference at *p* < 0.05, as determined by one-way ANOVA with Tukey’s post-test.

The spatiotemporal pattern of auxin response is crucial for plant leaf morphogenesis (Hay et al., 2006; Kawamura et al., 2010; Bilsborough et al., 2011; Kasprzewska et al., 2015). Previous studies revealed that the domain of auxin response in the tips of developing teeth of leaves of *epfl2* and multiple *er* family mutants becomes broader, suggesting that the EPFL2 peptide and ER-family proteins constitute ligand-receptor pairs that repress auxin responses in the growing leaf margin and thus determine the degree of leaf-tooth growth (Tameshige et al., 2016). To investigate the relationship between BIK1 function and auxin response during leaf-tooth growth, we analyzed the expression of the auxin response reporter *DR5pro::GFP* in *bik1* by confocal laser scanning microscopy. In the newly initiating leaves and the fully spreading new leaves at different growth stages of *bik1* plants, *DR5pro::GFP* fluorescence was restricted to the tip of a growing tooth (**Figures 5A–C**), and the GFP fluorescence signal in the leaf margins was weaker than that in Col-0 (**Figures 5D, E**) (GFP fluorescence intensity in the dashed rectangles in **Figures 5B, C** is quantified in **Figures 5D, E**), whereas in *er-105*, *DR5pro::GFP* expression extended laterally to the regions surrounding the leaf margin and growing tooth tip (**Figures 5A–C**), and the intensity of GFP fluorescence in the leaf margins was much stronger (**Figures 5D, E**). This observation suggests that BIK1 and ER have opposite functions in regulating auxin responses in developing leaf margins.

### BIK1 Interacts With ER-Family Proteins

**Figure 6:**
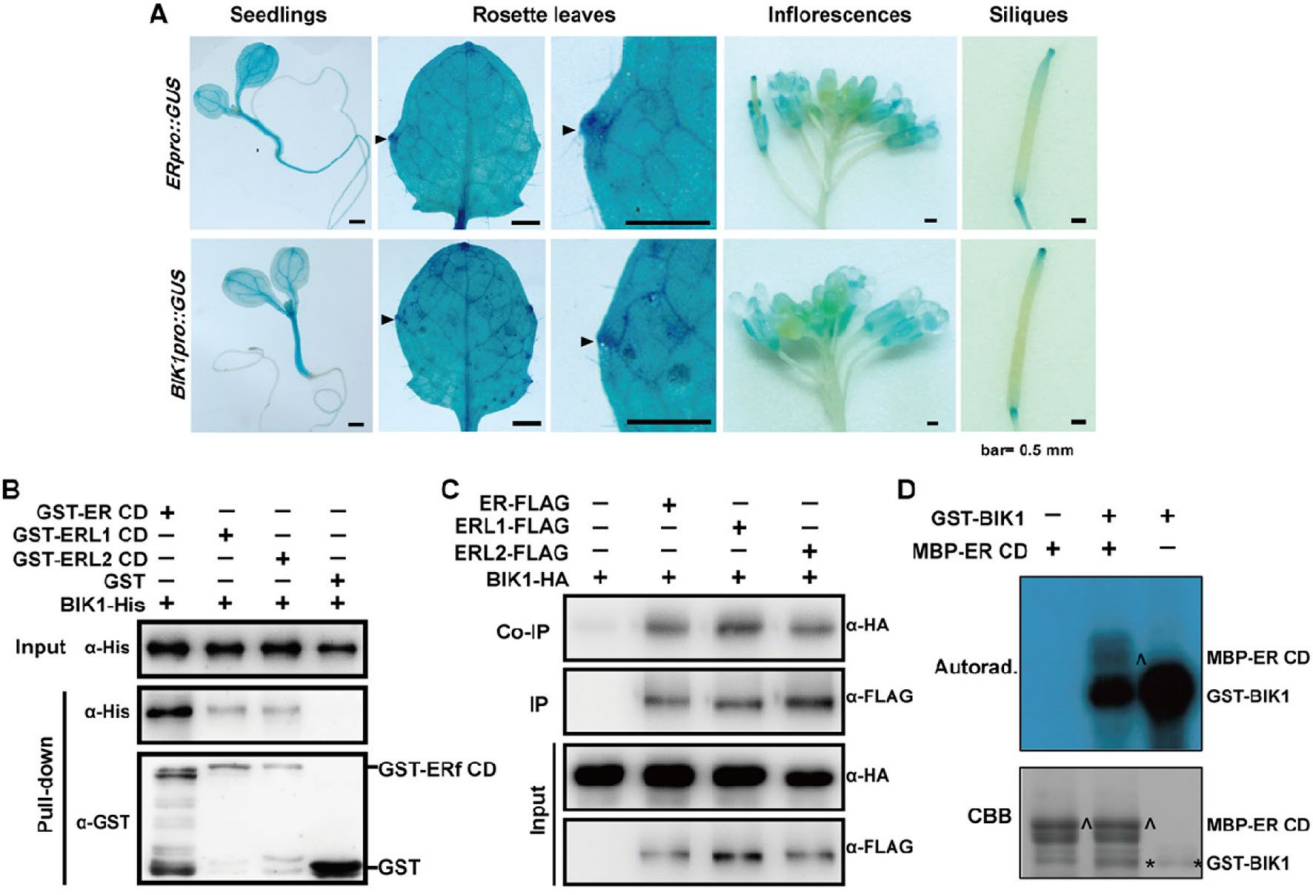
BOTRYTIS-INDUCED KINASE1 (BIK1) interacts with the ERECTA (ER) family. **(A)** GUS staining of 6-day-old seedlings, 4-week-old rosette leaves, 8-week-old inflorescences and siliques of *ERpro::GUS* and *BIK1pro::GUS* lines. Arrows indicate the growing tips of the leaf margins. Scale bar = 0.5 mm. **(B)** BIK1 interacted with the ER family in an *in vitro* pull-down assay. Total proteins were pulled down by glutathione Sepharose 4B and detected using an anti-His antibody. Experiments were repeated three times with similar results. **(C)** BIK1 interacted with the ER family in an *in vivo* coimmunoprecipitation assay. Protoplasts coexpressing ER/ERL1/ERL2-FLAG and BIK1-HA for 12 h were used for co-IP with anti-FLAG-coupled agarose. Experiments were repeated three times with similar results. **(D)** The *in vitro* phosphorylation assay of ER-CD by BIK1. Phosphorylation was detected by autoradiography (top), and the protein loading was shown by Coomassie brilliant blue (CBB) staining (bottom). Arrow indicated ER protein, and asterisk indicated BIK1 protein. Experiments were repeated three times with similar results.

Since ER and BIK1 play opposite roles in the regulation of leaf morphogenesis and inflorescence architecture in *A. thaliana*, we were curious about whether BIK1 is functional related to ER. Thus, we expressed GUS (*β*-glucuronidase) driven by either the *ER* promoter (*ERpro::GUS*) or the *BIK1* promoter (*BIK1pro::GUS*) in Col-0 plants. GUS staining assay revealed that both *ERpro::GUS* and *BIK1pro::GUS* were expressed in young seedlings and in the growing tips of the leaf margins, inflorescences, and siliques (**Figure 6A**), suggesting that *BIK1* and *ER* share similar spatial expression patterns in plants.

RLCKs in the cytoplasm often complex with RLKs and act as downstream components in different signal pathways. ER functions synergistically with its homologs, *ERL1* and *ERL2* in various aspects including plant development and responses to environmental changes. To test whether BIK1 directly interacts with ER-family proteins, we performed pull-down assay and *in vivo* co-IP assay. Both pull-down and co-IP assay confirmed that BIK1 can interact with ER, ERL1, and ERL2 (**Figures 6B, C**).

## Discussion

In plants, many RLCKs complex with RLKs and act as downstream components in the activation, transmission, and integration of intracellular signals (Macho and Zipfel, 2014). For example, BIK1 plays a positive role in immune signaling and a negative role in BR signaling (Lu et al., 2010; Lin et al., 2013; Li et al., 2014). Notably, *bik1* displayed abnormal developmental phenotypes, including serrated leaf margins and wrinkled surfaces, weaker stem strength, loose inflorescences architecture, reduced fertility, and smaller siliques (Veronese et al., 2006). However, how BIK1 functions in developmental processes remains unclear. In BR signaling, BIK1 inhibits the activation of BRI1 and thus plays a negative role in BR signal response (Lin et al., 2013). While loss of BRI1 function in *bri1-5 bik1* or *bri1-119 bik1* double mutant did not significantly affect the leaf margin and inflorescence development phenotypes observed in *bik1* (Lin et al., 2013), suggesting that these phenotypes were not mediated by BR signaling. In addition, *bik1* mutant exhibited a constitutive immune response, characterized by the increased expression of defense-related genes, such as *PATHOGENESIS-RELATED PROTEIN1* (PR1), *PHYTOALEXIN DEFICIENT4* (*PAD4*), and *ETHYLENE RESPONSE FACTOR1* (ERF1), accompanied with salicylic acid accumulation in the leaves (Lei et al., 2014). However, the SA accumulation deficient mutant *sid2-2* and resistance protein-deficient mutant *pad4-1* inhibited downstream immune responses induced by *bik1* mutations, without affecting leaf and inflorescence development (Liu et al., 2017). This indicates that the developmental defects of *bik1* are not directly related to the constitutive activation of immune responses.

In the current study, we designed a forward genetic screen by EMS mutagenesis based on modified developmental phenotypes of *bik1*. We successfully identified two mutant lines both rescued the defective phenotypes of *bik1*, named as *mobd1 bik1* and *mobd2 bik1*, respectively (**Figures 1A, B**). Using a whole-genome SNP-based deep sequencing technique, we identified several candidates from the *mobd1* and *mobd2* group mutants. Among them, *ER* gene represented a good candidate which were selected and confirmed by resequencing (**Figure 1C**). The loss-of-function mutant of BIK1 displayed abnormal phenotypes, such as wrinkled leaves with serrated margins (Veronese et al., 2006), loose inflorescences with elongated pedicels, and longer and much narrower silique tips (**Figures 2**–**4**). By contrast, *er* mutant exhibited toothless leaves (**Figure 4**) and a compact inflorescence with short pedicels and short, broad silique tips (**Figures 2** and **3**). Furthermore, loss-of-function of BIK1 and ER had opposite effects on auxin responses in developing leaf margin (**Figure 5**), thus made the different leaf morphogenesis of *bik1* and *er* mutants. Our data suggest that ER and BIK1 might perform opposite functions in plant development and that the phenotypes of the *bik1* mutant can be rescued by mutation of *ER*.

Notably, BIK1 performs signal transduction through subtle mechanisms downstream of different RLKs. In flg22-induced immune response, BIK1 dissociates from FLS2 complex and activates the downstream effectors upon flg22 treatment (Lu et al., 2010; Zhang et al., 2010; Li et al., 2014). Similarly, chitin triggers dissociation between BIK1 and chitin elicitor receptor kinase 1 in the chitin-triggered immune response (Liu et al., 2018). BIK1 negatively regulates BR signaling *via* a specific molecular mechanism: in the absence of BR treatment, BIK1 associates with BRI1 and inhibits the initiation of BR signaling, following BR treatment, BIK1 is released from BRI1 to transduce BR signaling (Lin et al., 2013). In the current study, we found that BIK1 physically interacts with ER-family proteins (**Figures 6B, C**). In addition, *in vitro* kinase assay showed that the CD of ER (ER-CD) could be phosphorylated by BIK1 (**Figure 6D**), indicating a transphosphorylation modification of ER by BIK1 protein. For the strong autophosphorylation of BIK1, we performed an *in vitro* kinase assay with ER-CD and the kinase dead mutant BIK1. However, we did not observe the phosphorylation of BIK1 by ER (data not shown). Considering that *er bik1* double mutants possessed a rescue or partial rescue of phenotypes in internodes development, leaf morphogenesis, and inflorescence architecture compared to *er* mutant plants (**Figures 2**–**5**), the potential relationship between BIK1 and ERECTA family should be paid attention to.

Moreover, a recent study demonstrated that two members of BRASSINOSTEROID SIGNALING KINASE family, BSK1 and BSK2, function upstream of YDA and take part in the ERECTA/YDA pathway (Neu et al., 2019). The *bsk1 bsk2* double mutants exhibited similar phenotypes to *yda* or *erf* mutants, suggesting a broadly overlapping role of BSK1/2 in BR and ER pathways. Therefore, whether BIK1 plays a negative role in ER signaling, as its function in BR signal pathway, and the detailed mechanism of this event is worthy of further research.

Taken together, although the direct signal transduction and functional relationship between ER-family and BIK1 is still unverified, a potential correlation between BIK1 and ER signal pathway should be paid attention to. Future studies should focus on whether BIK1 also functions as a regulator in ER signaling pathway, which maybe dynamically interact with ER upon treatment with EPFL2 or EPFL/4/6, the peptide ligands of ER that modulate leaf and inflorescence architecture, respectively. Furthermore, whether BIK1 correlates with the downstream components in ER signal, including YDA-dependent, or other undiscovered pathways is still unclear. These potential molecular mechanisms warrant further investigation.

## Data Availability Statement

The datasets generated for this study are available on request to the corresponding author.

## Author Contributions

JL, H-BW, and BL designed the study. SC and JL performed research. YL, LC, TS, NY, and H-BW analyzed data. BL, SC, and JL wrote the article.

## Funding

This study was supported by the National Natural Science Foundation of China (No. 31571249, No. 31425003) and the project of Sun Yat-sen University (33000-31143406).

## Conflict of Interest

The authors declare that the research was conducted in the absence of any commercial or financial relationships that could be construed as a potential conflict of interest.
